# Supplementary material for: Physical Fitness and Performance in Talented & Untalented Young Chinese Soccer Players
Source: Healthcare (Basel). 2022 Jan 4;10(1):98. doi: 10.3390/healthcare10010098 (PMC8775658; doi:10.3390/healthcare10010098)
Supplement: Supplementary file 1 [file healthcare-10-00098-s001.zip › healthcare-1506456-supplementary.pdf]

SUPPLEMENTARY DOCUMENTATION

Technical and tactical aspects analyzed by the Evergrande Football School Spanish team of expert coaches

In order to divide the group of young players into two groups of similar sporting level (talented and untalented), before the start of each season, the EFS technical staff visualizes a set of matches from the immediately preceding sport season. The coaches do this on the basis of technical and tactical performance indicators that are applied to each player individually. They use an observation template in which they categorize a series of variables. Once all players have been scored, they calculate the Z-value of each player [(player score - average score of the whole group) / standard deviation of the whole group]. Players who are at or above 1.5 standard deviations are considered talented.

Below is the template used by the coaches with the corresponding variables and the scoring system applied with two examples for each sport levels: "talented: ≥ 1.5 sd" and "untalented: <1.5 sd".

	**Technical skills (50%)**														
	**Passes (n)**		**Successful Passes (type)**			**Ball control actions (n)**		**Direct kick Shots (n)**		**Technical actions (n)**				**Overall technical**
										Offensive		Defensive		
**Id.**	Successful	Unsuccessful	One-touch	Long	Piercing / Goal	Successful	Unsuccessful	Successful	Unsuccessful	Successful	Unsuccessful	Successful	Unsuccessful	
**20013**	93	12	82	6	5	53	7	3	2	34	12	37	6	**181,0**
**70040**	73	6	70	3	0	70	3	2	2	42	3	41	4	**210,0**
**40080**	41	14	39	1	1	21	9	0	0	32	12	24	11	**72,0**
**70155**	31	12	43	1	2	19	17	0	1	30	12	24	1	**61,0**

	**Tactical skills (50%)**														
	**Game spaces management**								**Ball possession management**						
	**Offensive and creative game**				**Defensive game**				**Offensive and creative game**						
**Id.**	Unmarking (n)	Generation Passing line (n)	Offside (n)	Tactical Errors (n)	Marking (n)	Block Passing line (n)	Forced Offside (n)	Tactical Errors (n)	Conduction entering the goalkeeper's area (n)	Conduction between transition zones (n)	1*vs*1 successful actions (n)	1*vs*1 unsuccessful actions (n)	Shots on goals excepted direct kick shots (n)	Goals excepted direct kick shots (n)	**Overall tactical**
**20013**	37	34	3	4	26	9	0	3	28	27	33	6	3	3	**184,0**
**70040**	57	35	0	6	32	19	2	2	25	26	31	8	0	0	**211,0**
**40080**	24	6	3	6	4	21	1	3	14	13	13	11	5	2	**80,0**
**70155**	8	19	3	10	11	19	3	8	17	15	18	7	1	0	**83,0**

	**Classification as talented or untalented according to the criteria of the Evergrande Football School Spanish coaches**						
**Id.**	**Technical Skills**	**Tactical Skills**	**Overall (Technical + Tactical)**	**All group (average ± sd) (n=722)**		**Z-value (≥1,5=Talented)**	
**20013**	181	184	365	211,7	101,9	**1.5**	Talented
**70040**	210	211	421			**2.1**	Talented
**40080**	72	80	152			**-0.6**	Untalented
**70155**	61	83	144			**-0.7**	Untalented
